# Novel Pixelwise Co-Registered Hematoxylin-Eosin and Multiphoton Microscopy Image Dataset for Human Colon Lesion Diagnosis

**DOI:** 10.1016/j.jpi.2022.100012

**Published:** 2022-02-07

**Authors:** Artzai Picon, Elena Terradillos, Luisa F. Sánchez-Peralta, Sara Mattana, Riccardo Cicchi, Benjamin J. Blover, Nagore Arbide, Jacques Velasco, Mª Carmen Etzezarraga, Francesco S. Pavone, Estibaliz Garrote, Cristina L. Saratxaga

**Affiliations:** a TECNALIA, Basque Research and Technology Alliance (BRTA), Astondo bidea, Edificio 700, 48160 Derio (Bizkaia), Spain; b University of the Basque Country UPV/EHU, Ingeniero Torres Quevedo Plaza, 1, 48013 Bilbao, Spain; c Centro de Cirugía de Mínima Invasión Jesús Usón, Carretera N-521, km. 41,8, 10071 Cáceres, Spain; d National Institute of Optics, National Research Council (CNR-INO), Largo E. Fermi 6, 50125 Florence, Italy; e European Laboratory for Non-Linear Spectroscopy (LENS), Via N. Carrara 1, Sesto Fiorentino 50019, Italy; f Department of Surgery and Cancer, Imperial College London, London, UK; g Osakidetza Basque Health Service, Basurto University Hospital, Department of Pathological Anatomy, Bilbao (Bizkaia), Spain; h Department of Physics, University of Florence, Via G. Sansone 1, 50019 Sesto Fiorentino, Italy

**Keywords:** Multiphoton Microscopy (MPM), Dataset, Optical Biopsy, Convolutional Neural Network (CNN), Colorectal Polyps

## Abstract

Colorectal cancer presents one of the most elevated incidences of cancer worldwide. Colonoscopy relies on histopathology analysis of hematoxylin-eosin (H&E) images of the removed tissue. Novel techniques such as multi-photon microscopy (MPM) show promising results for performing real-time optical biopsies. However, clinicians are not used to this imaging modality and correlation between MPM and H&E information is not clear. The objective of this paper is to describe and make publicly available an extensive dataset of fully co-registered H&E and MPM images that allows the research community to analyze the relationship between MPM and H&E histopathological images and the effect of the semantic gap that prevents clinicians from correctly diagnosing MPM images. The dataset provides a fully scanned tissue images at 10x optical resolution (0.5 µm/px) from 50 samples of lesions obtained by colonoscopies and colectomies. Diagnostics capabilities of TPF and H&E images were compared. Additionally, TPF tiles were virtually stained into H&E images by means of a deep-learning model. A panel of 5 expert pathologists evaluated the different modalities into three classes (healthy, adenoma/hyperplastic, and adenocarcinoma). Results showed that the performance of the pathologists over MPM images was 65% of the H&E performance while the virtual staining method achieved 90%. MPM imaging can provide appropriate information for diagnosing colorectal cancer without the need for H&E staining. However, the existing semantic gap among modalities needs to be corrected.

## Introduction

Colorectal cancer ranks as one of the predominant cancers, being the third most commonly occurring cancer in men and the second most commonly occurring cancer in women.^1^ Fortunately, its early detection significantly increases the survival rate, reaching a cure rate of 90% when diagnosed at a localized stage.^2^^,^^3^ Moreover, colorectal cancer can be prevented by the early detection of polyps that might progress towards cancer. 20–40% of patients present polyps and traditional colonoscopies present average adenoma missing rates that can range from 12.5% to 68.1% but that can be reduced by using new technologies.^4^ Furthermore, despite 29–42% of the detected polyps being hyperplastic with no malignant risk, the rest corresponds to neoplastic tissue that can progress to colorectal cancer if not removed.^5^, ^6^, ^7^ Common practice involves removal of the identified polyps, followed by histopathological analysis. Besides this, during a conventional colon polypectomy using endoscopic mucosal resection (EMR), residual adenomatous tissue rates of 46% and postprocedure recurrence rates of 12–21.9% have been reported.^8^, ^9^, ^10^ This implies that follow-up and reinterventions are necessary. This affects the prognosis of the patient while increasing the risk of complications such as bleeding or perforation. Therefore, optical biopsy based on imaging technologies that generates images of the cellular structure of polyps aids real-time clinical decision-making.^10^

Already in 2017, Byrne et al.11 identified that computer-aided detection (CAD) systems have great potential in colonoscopy on three key areas: adequacy of mucosal inspection, polyp detection, and optical biopsy. These CAD systems have recently been boosted with the great success of artificial intelligence and deep learning, giving place to an exponential growth of works related to detection, localization, and optical biopsy of polyps,^12^ mainly based on traditional image modalities, namely white light imaging, narrow band imaging, or hematoxylin–eosin (H&E).^13^^,^^14^ To this end, extensive research is being done on the different key aspects of deep learning models to improve performance: architectures,^15^^,^^16^ loss functions,^17^^,^^18^ augmentation techniques^19^^,^^20^, or dataset generation.^21^^,^^22^

Because of all these aspects, deep learning models based on novel technologies such as reflectance confocal microscopy, multiphoton microscopy (MPM), or optical coherence tomography (OCT) among others are being analyzed to allow performing new in-situ and in-vivo diagnostic by measuring the presence and degree of malignancy for the identified tissue.^23^ This will allow safer resection with clean margins, as the polyp margins could be analyzed prior and after resection.

In the last couple of decades, two-photon fluorescence (TPF) and second-harmonic generation (SHG) microscopy have been largely used in biomedical field. They intrinsically offer several advantages with respect to other optical techniques, like wide-field and confocal microscopy, such as reduced photo-damage/photo-toxicity, optical sectioning capability, reduced scattering, and high-resolution deep-tissue imaging.

In this sense, recent studies^24^^,^^25^ conclude that images of human colon tissue obtained with MPM at high resolution (40× objective with 1.3 NA and 195 nm/pxl, 25x objective with 1.1 NA, respectively) contain morphological and functional information for discriminating between cancer, adenoma, and normal tissue. A more recent work^1^ has validated that the information contained on MPM images can be used to successfully build machine learning models that can accurately distinguish among malignant neoplastic and non-malignant tissue. However, it presents mainly two limitations:

(1.) These novel techniques are unfamiliar for clinicians and face barriers for being incorporated into clinical practice.
(2.) There is a lack of abundant labeled images that are required for modern machine learning models to appropriately learn the discriminative features for novel modalities.^26^^,^^27^

To overcome this, it is necessary to develop machine learning methods for converting images from the novel domain (e.g., MPM) into the known gold-standard domain (H&E). To this end, we proposed^28^ an algorithm to virtually transform an MPM image into its corresponding H&E counterpart by using simulated images and demonstrating the transformation was affordable by current algorithms.

In the current work, we describe and make publicly available a real MPM and H&E colon tissue dataset with pixel correspondence among both modalities. This dataset should serve the research community to deepen its knowledge on these techniques both from the clinical point of view and the algorithmic point of view.

Additionally, to give an insight on the capabilities for clinicians to diagnose over MPM images, we analyze the relative capability of clinicians to diagnose image parts of MPM images compared to H&E image parts. Finally, we also measure the relative capability of clinicians to diagnose image parts of MPM images that have been virtually converted into H&E image parts by means of the method we proposed in ^28^.

## Materials and Methods

### Dataset Definition

The dataset consists of an extension of the dataset we presented in ^1^. A set of 50 samples obtained during colonoscopies and colectomies carried out between the years 2012 and 2017 at the Basurto University Hospital (Spain). These are 24 malignant neoplasms (adenocarcinomas), 19 preneoplastic lesions (adenomas), 2 hyperplasia samples, and 5 healthy tissues, obtained from 24 men and 19 women. The samples were diagnosed by the Pathological Anatomy Department and the FFPE (formalin-fixed paraffin-embedded) blocks were stored in the Basque Biobank (structure accredited by the Health Department and inscribed in the register of the Instituto de Salud Carlos III). All the samples were processed and sliced after signing informed consent without altering the standard clinical procedures. The sliced samples were scanned at the joint-lab between National Institute of Optics and European Laboratory for Non-linear Spectroscopy in Florence, Italy, using a custom-made multiphoton microscope for co-registered two-photon fluorescence (TPF) and second harmonic generation (SHG) microscopy,^29^^,^^30^ and later stained with H&E (hematoxylin & eosin).

The different image modalities were reconstructed and co-registered by performing non-rigid deformation allowing pixel correspondence among the different modalities. Pathologists manually labeled the regions where lesions were present. The dataset provides fully scanned tissue images at 10× optical resolution (0.5 μM/px).

The results are shown in Table 1 accordingly to the nomenclature specified in ^31^. In the case of adenocarcinomas, the terms "low grade" and "high grade" refer to the tumor grading. Low includes the well and moderately differentiated grades, whereas high refers to the poorly differentiated grade. This table might slightly differ from ^1^ as new sections were cut from the FFPE blocks with 10 μM thickness to allow simultaneous MPM and H&E scanning of the same block.

**Table 1:** Dataset histopathological description

Sample Id.	Slide content description	Histological analysis	Scanned tissue sections	Co-registered image resolution (px)
56	2.2 cm part of a 7 cm size polyp obtained from the descending colon	Villous adenoma with high grade dysplasia	1	43397×44426
56_SA	Healthy tissue adjacent to id 56 sample	Healthy	1	29950×19867
57	1 cm part of a 3.7 cm size polyp obtained from the ascending colon	Tubulovillous adenoma with high grade dysplasia	1	31531×30663
57_SA	Healthy tissue adjacent to id 57 sample	Healthy	1	26308×16938
58	2.3 cm part of a 4 cm size polyp obtained from the descending colon	Villous adenoma with high grade dysplasia	1	66293×31593* Due to size, it is saved as png
58_SA	Healthy tissue adjacent to id 58 sample	Healthy	1	15914×9927
59	0.4 cm size polyp obtained from the ascending colon	Tubular adenoma	2	5054×8994
60	3.3 cm size polyp obtained from the ascending colon	Tubulovillous adenoma with high grade dysplasia	2	52721×42094
61	2.1 cm part of a 9 cm size polyp obtained from the descending colon	Villous adenoma with high grade dysplasia	1	51197×42469
62	0.5 cm size polyp obtained from the ascending colon	Tubular adenoma	1	28032×25116
63-1	1.1 cm part of a 2.8 cm size polyp obtained from the descending colon	Tubular adenoma with low grade dysplasia	1	55092×43338
63-2	1.65 cm part of a 2.8 cm size polyp obtained from the descending colon	Adenocarcinoma over tubulovillous adenoma with high grade dysplasia	1	51503×41160
64	0.9 cm part of a 1.2 cm size polyp obtained from the ascending colon	Tubular adenoma with low grade dysplasia	1	31370×32749
65	6 polyps with sizes between 0.32 and 0.54 cm, belonging to a case of 118 polyps with sizes between 0.6 and 6 cm, obtained from the ascending colon	Tubular adenoma with low grade dysplasia	1	40623×38695
66	3.1 cm part of a 9 cm size polyp obtained from the ascending colon	Tubulovillous adenoma with high grade dysplasia	1	61086×40007
67	1.4 cm size polyp obtained from the ascending colon	Sessile tubular adenoma, low grade	1	59641×43416
68	0.2 cm part of a 0.3 cm size polyp obtained from the descending colon	Tubular adenoma with low grade dysplasia	1	43804×28848
69-1	2 polyps with sizes of 0.2 and 0.3 cm, belonging to a case of 5 polyps, obtained from the descending colon	Hyperplastic polyp	1	9849×9149
69-2	0.36 cm part of a 0.4 cm size polyp obtained from the descending colon	Tubular adenoma with low grade dysplasia	1	42197×37311
70	0.8 cm part of a 1 cm size polyp obtained from the ascending colon	Tubular adenoma with low grade dysplasia	1	32229×35769
71	2.2 cm part of a 2.5 cm size polyp obtained from the ascending colon	Tubulovillous adenoma with high grade dysplasia	1	40979×39476
72	3.2 cm part of a 4 cm size polyp obtained from the ascending colon	Tubular adenoma with low grade dysplasia	1	64851×39618
73	0.2 cm size polyp obtained from the descending colon	Hyperplastic polyp	1	45722×42651
74	1.2 cm part of a 1.8 cm size polyp obtained from the descending colon	Tubulovillous adenoma	1	25634×31466
75	no polyp from a case with a 3 cm size polyp obtained from the descending colon	Invasive colloid adenocarcinoma	1	34434×33708
76	0.4 cm part of a 0.6 cm size polyp obtained from the transverse colon	Tubular adenoma	6	14074×13439
77	No polyp obtained from the ascending colon	Low grade adenocarcinoma,Not Otherwise Specified ( NOS)	1	41459×41225
77_SA	Healthy tissue adjacent to id 77 sample	Healthy	1	35964×28569
78	2.2 cm part of a 3 cm size polyp obtained from the transverse colon	Low grade adenocarcinoma, NOS over high grade tubulovillous adenoma	1	55961×40668
79	No polyp obtained from the ascending colon	Low grade adenocarcinoma, NOS	1	65085×37985
80	No polyp obtained from the descending colon	Low grade adenocarcinoma, NOS	1	57283×30663
80_SA	Healthy tissue adjacent to id 80 sample	Healthy	1	23315×23950
81	0.6 cm size polyp obtained from the transverse colon	Low grade adenocarcinoma, NOS	1	49701×18623
82	1.4 cm part of a 4 cm size polyp obtained from the descending colon	Low grade adenocarcinoma, NOS	1	28693×29872
83	2 cm part of a 2.3 cm size polyp obtained from the descending colon	Low grade adenocarcinoma, NOS	1	44997×39411
84	2.6 cm part of a 4 cm size polyp obtained from the descending colon	Low grade adenocarcinoma, NOS	1	54380×40279
85	1.5 cm part of a 2.5 cm size polyp obtained from the ascending colon	Low grade adenocarcinoma, NOS	1	59914×43532
86	1.2 cm part of a 1.5 cm size polyp obtained from the descending colon	Low grade adenocarcinoma, NOS	1	39877×32698
87	1.6 cm part of a 2.6 cm size polyp obtained from the ascending colon	Low grade adenocarcinoma, NOS	1	51396×43066
88	1.9 cm part of a 4.5 cm size polyp obtained from the ascending colon	Low grade adenocarcinoma, NOS	1	60367×39566
89	1.9 cm part of an 8.7 cm size polyp obtained from the descending colon	Low grade adenocarcinoma, NOS	1	50807×25518
90	1.6 cm part of a 3.5 cm size polyp obtained from the descending colon	Low grade adenocarcinoma, NOS	1	46513×39411
91	1.8 cm part of a 6.5 cm size polyp obtained from the descending colon	Low grade adenocarcinoma, NOS	1	60212×38076
92	2.7 cm part of an 8 cm size polyp obtained from the transverse colon	High grade adenocarcinoma NOS	1	58877×37752
93	1 cm part of an 8 cm size polyp obtained from the descending colon	High grade adenocarcinoma, NOS	1	34318×37558
94	2.4 cm part of a 6 cm size polyp obtained from the ascending colon	High grade adenocarcinoma, NOS	1	48885×44478
95	1.3 cm part of a 4 cm size polyp obtained from the descending colon	Low grade adenocarcinoma, NOS	1	42884×26645
96	1.7 cm part of a 5 cm size polyp obtained from the descending colon	Low grade adenocarcinoma, NOS	1	49014×40253
97	1.3 cm part of a 5 cm size polyp obtained from the descending colon	High grade adenocarcinoma, NOS	1	46811×40020
98	2 cm part of a 5 cm size polyp obtained from the descending colon	High grade adenocarcinoma, NOS	1	42268×41031

The dataset is openly available at https://www.biobancovasco.org/en/Sample-and-data-e-catalog/Databases/PD181-PICCOLO-EN3.html and can be downloaded after filling in a request form.

### Acquisition Procedure

Biological tissues can be imaged by TPF microscopy because cells and extracellular matrix intrinsically contain a variety of fluorescent molecules without the needs of exogenous labels.^33^ The light emitted by the sample can be collected by a photomultiplier to produce a fast imaging of the specimen under analysis. In this setup, using the same NIR laser source, SHG microscopy can be also performed, in order to obtain additional morphological information concerning non-centrosymmetric molecular structures, such as collagen fibers.^34^ Both TPF and SHG signals can be collected at the same time by separating them using an optical filter.

**Fig. 1:**
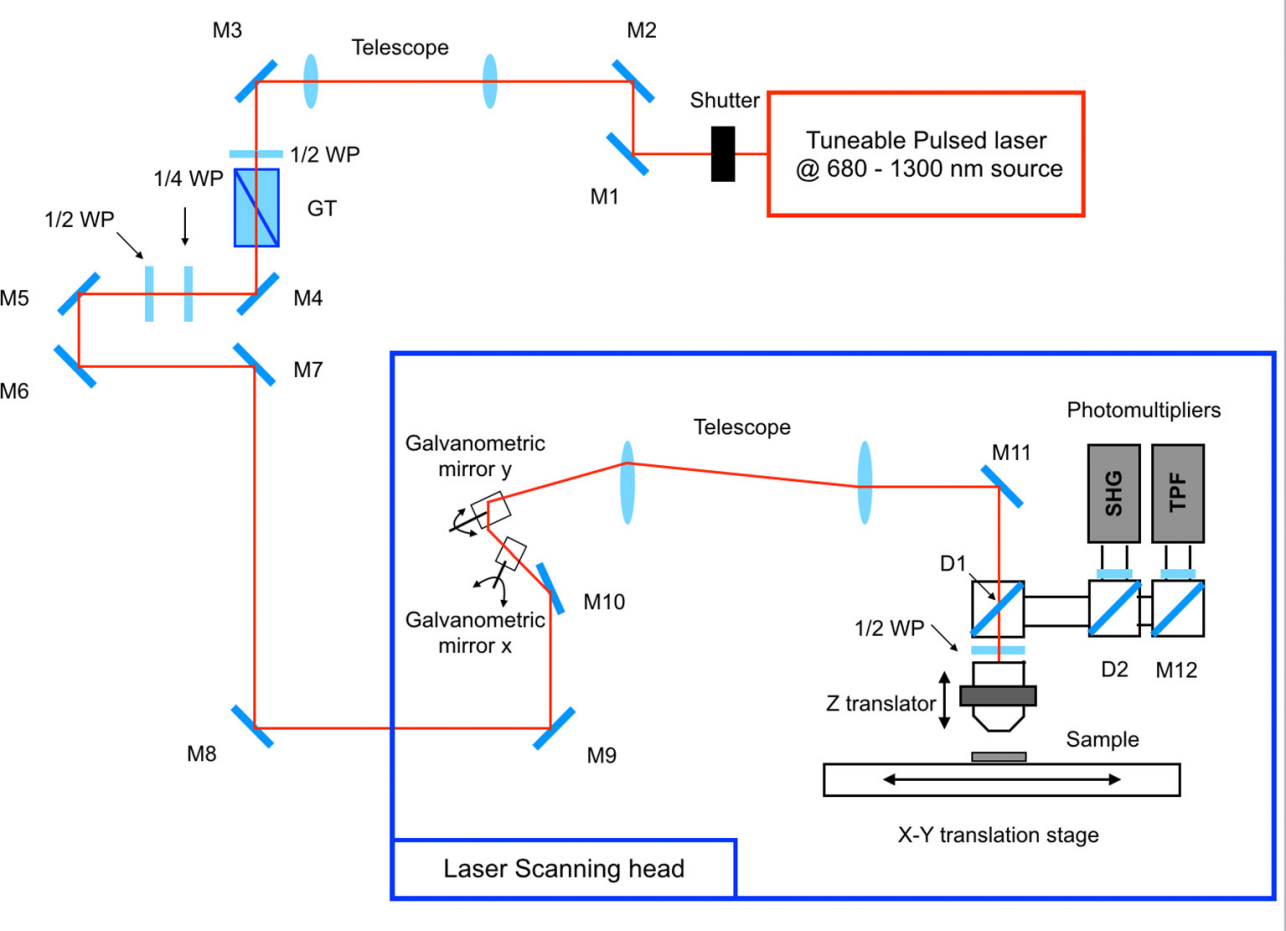
Schematic of the custom-made multimodal multiphoton microscope: tuneable source; shutter; mirrors (M); telescope lenses; half wave plate (1/2WP); quarter wave plate (1/4WP); Glan-Taylor polarizer (GT); galvanometric mirrors (x, y); scan lens and tube lens (telescope); objective translator (Z translator); XY-translation stage ; dichroic mirror (D), SHG and TPF photomultipliers.

The experimental setup used for the acquisition is similar to the one described in ^1^. It consists of a custom-made multimodal multiphoton microscope, whose optical scheme is shown in Fig. 1. The excitation source for multiphoton imaging is a Chameleon Discovery (Coherent, Santa Clara, CA), an Yb-based femtosecond pulsed laser at 80 MHz rate with two synchronous outputs: the beam used in the experiments here described is tunable in the 680–1300 nm range and pulses of about 100 fs, the other beam with a fixed 1040 nm wavelength was not used in this experiment. The laser beam passes through a mechanical shutter, which minimizes the sample exposure to the laser light while acquiring images, through a telescope mounted for collimation and beam sizing. A motorized half-waveplate together with a Glan-Taylor polarizer are used for power dimming. After these optical elements, the laser light is then directed to the scanning system, consisting of a vertically mounted stainless-steel optical breadboard, placed onto an antivibration optical table (Thorlabs Inc., Newton, NJ). Two galvanometric mirrors (Cambridge Technology, Bedford, MA) provide fast beam scanning and tube and scan lenses optically relays the beam to the objective lens, mounted on an optomechanical support equipped with both mechanical and piezoelectric (P-725KHDS PIFOC, Physik Instrumente, Karlsruhe, Germany) translators for gross and fine movements, respectively. Spatial motions are performed by means of an xy translator (M-687 PIline, Physik Instrumente, Karlsruhe, Germany), in which the sample is placed, allowing mapping of large areas through movements over a broad range with submicrometric resolution. Backward emitted fluorescence and SHG signal from the sample is then collected by the same objective lens used for excitation after the separation from the excitation radiation through a dichroic mirror (FF665-Di02 – 25 × 36, Semrock Inc. New York, NY) placed into the first cube of the kinematic support. Another dichroic beam splitter (FF452-Di01, Semrock Inc. New York, NY) is located into the second kinematic mount and it is useful to split SHG and TPF signals, sending them to a two different photomultiplier tubes H7422-40 (Hamamatsu, Hamamatsu City, Japan) through their relative lenses. A large band-pass filter (FF01-505/119-25, Semrock Inc. New York, NY) is used for the detection of TPF signal, whereas a narrow band pass filter, spectrally centred at 386 ± 12 nm (FF01-386/23-25, Semrock Inc. New York, NY) is used to detect only the SHG signal. The photocurrent is integrated using custom electronics and acquired on a PC through an acquisition board PCI-MIO (National Instruments, Austin, TX) that allows synchronous signal sampling and scanner driving. System control and data acquisition are controlled using a custom software developed using LabVIEW 2015 (National Instruments, Austin, TX) development module. A more detailed description of the experimental setup can be found in literature.^29^^,^^36^

**Fig. 2:**
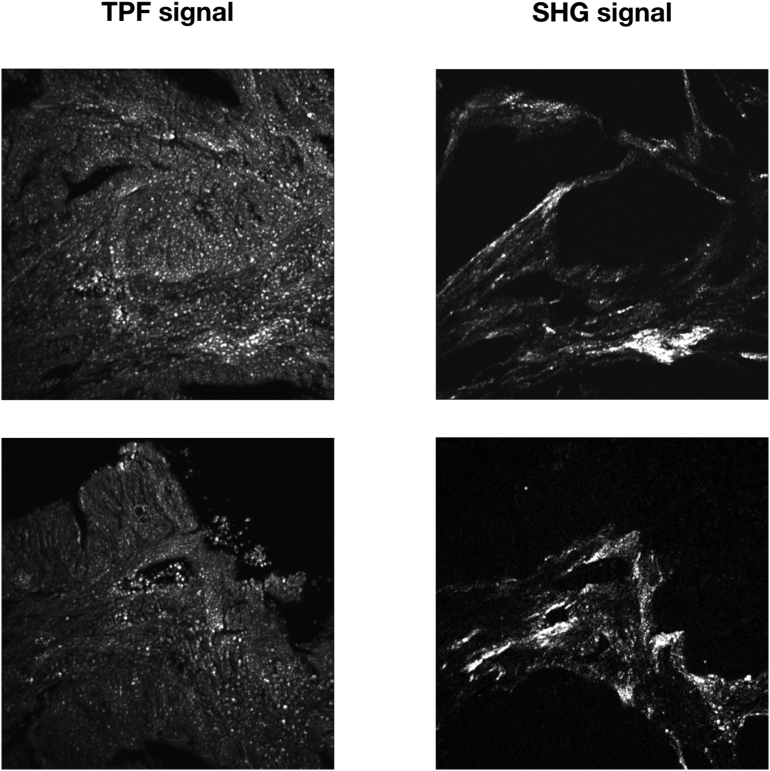
Individual image tiles acquired using TPF microscopy in different positions of a 10 μM thick paraffin-embedded tissue slide with sample 98 and the correspondent SHG image diagnosed as high-grade adenocarcinoma. The images show cells with different shape and morphology acquired in different regions of the sample, demonstrating the capability of TPF microscopy for the label-free morphological assessment of tissues. Each image is 511 × 511 μM^2^ with a resolution of 1024 × 1024 pixel^2^.

Multiphoton fluorescence and SHG images were acquired using an excitation wavelength of 785 nm, focused on the sample by means of a Plan-Apochromat 10× objective lens (NA 0.45, WD 2.1 mm, Carl Zeiss Microscopy, Jena, Germany). Image tiles were acquired using a field of view of 511 × 511 μM, a resolution of 1024 × 1024 pixels, a pixel dwell time of 5 μsec, and an average laser power of about 20 mW on the sample. As an example of captured images, Fig. 2 shows several 511 × 511 μM image tiles acquired with the multiphoton microscope using TPF and the corresponding SHG images in different positions of the same tissue slide.

**Fig. 3:**
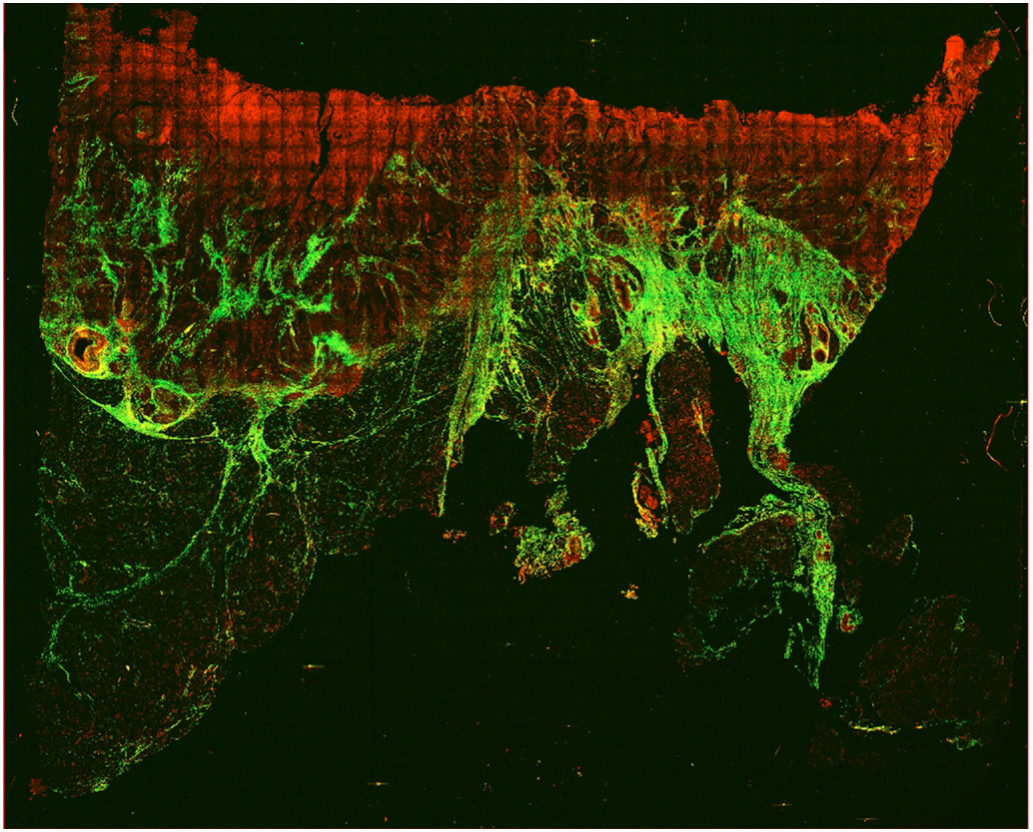
Merged image of TPF (red) and SHG (green) of a whole 10 μM thick paraffin-embedded tissue slide with sample 98 diagnosed as high-grade adenocarcinoma. The signal originates mainly from mitochondrial NADH in the cell cytoplasm and from elastic fibers and other fluorescent molecules in the extracellular matrix. This image has been obtained by concatenating 48 by 39 image tiles, resulting in an overall field of view of 24.528 × 19.929 mm.

Fig. 3 shows an example of a whole 10 μM thick paraffin-embedded tissue slide scanned with the multiphoton microscope. The image has been generated by concatenating all the individual TPF (red) and SHG (green) image tiles. TPF and SHG images were merged together with different colours in order to overlap the two images and underline the different contributions supplied by the two techniques.

Samples analyzed for this study were cut with a rotary microtome (RM2255, Leica Biosystems, Wetzlar, Germany) at 10 μM from Formalin-Fixed Paraffin-Embedded (FFPE) blocks of human tissue. 10 μM was selected as optimal thickness to allow simultaneous scanning of the samples to have enough depth for the MPM microscopy scanning as well as to avoid sticking and tissue damage on the slide scanner. Then, superfrost slides (LineaLAB, Badalona (Barcelona), Spain) were H&E stained in an automated slide stainer (SIMPHONY system, Roche Diagnostics, Basel, Switzerland). The histopathologists analyzed the stained slides under a microscope and annotated them as detailed on Section 2.3. Finally, all the slides were scanned with a fully motorized microscope (BX61, Olympus Corporation, Tokyo, Japan) equipped with Ariol software platform, where all the images had 20× magnification.

### Dataset Processing and Labeling

**Fig. 4:**
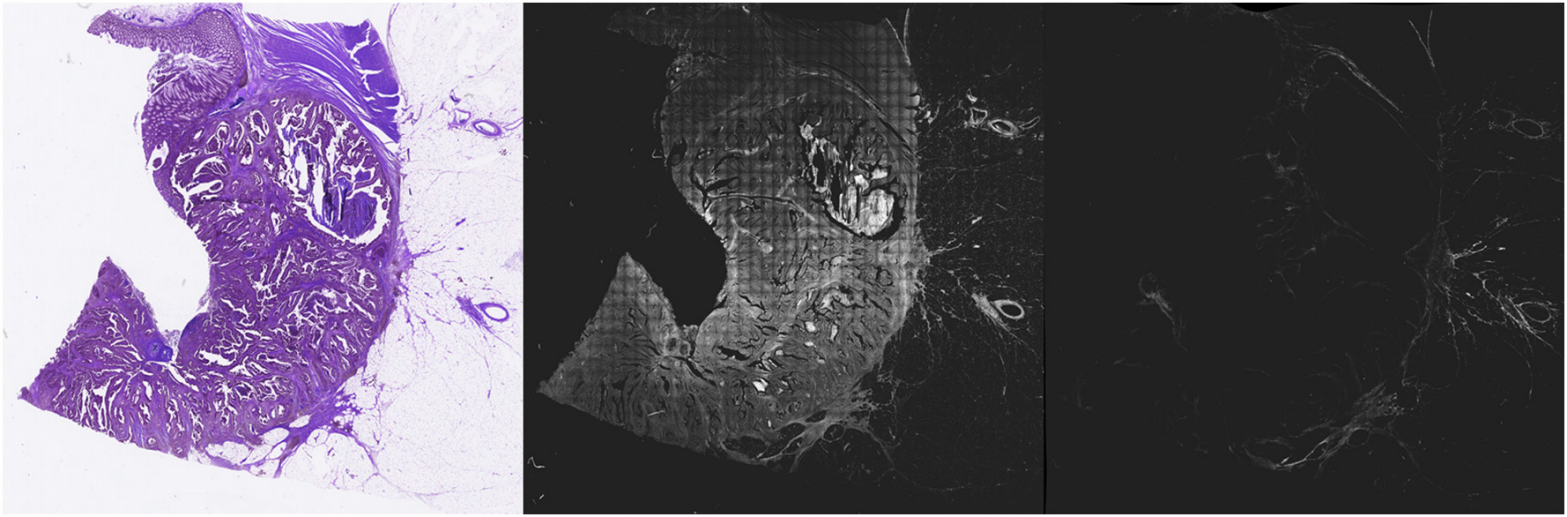
Acquired full tissue reconstruction from a 10 μM thick paraffin-embedded tissue slide from sample 90 diagnosed as low-grade adenocarcinoma. Left) H&E image, middle) TPF image, right) SHG image.

The tiles obtained by the H&E acquisition were aggregated in order to create a high-resolution image of the whole tissue. In a similar way, MPM acquired tiles, both for TPF and SHG, were also aggregated into single high-resolution images. Fig. 4 shows a reconstructed image for H&E, TPF, and SHG contrast mechanisms.

**Fig. 5:**
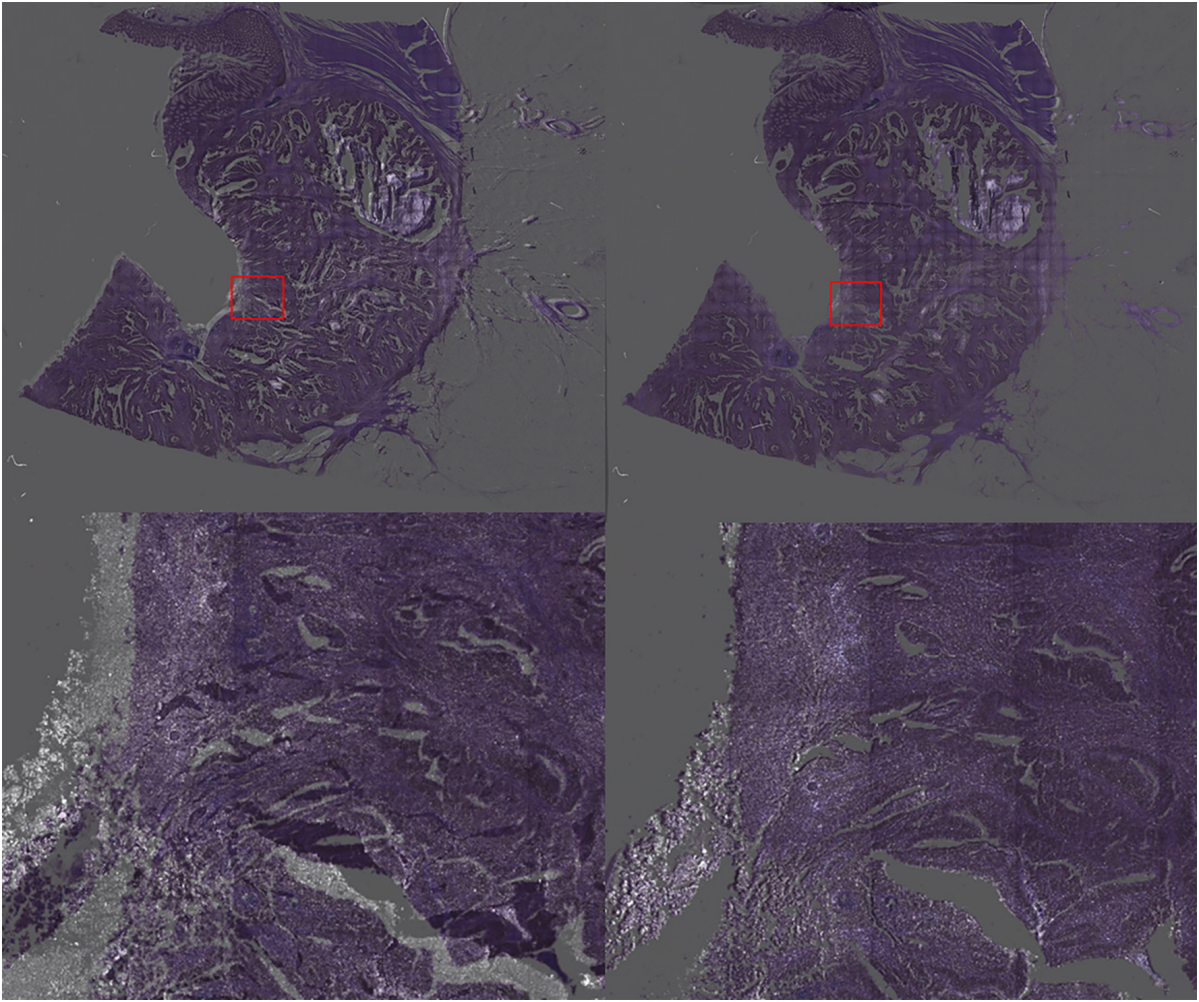
Effect of the co-registration process. Both TPF and H&E images are overimposed to appreciate the deformation error. Left) Overimposed images after the rigid co-registration method. Right) Overimposed images after the deformable co-registration method. Details can be appreciated on the bottom.

From each of these images, a rectangle showing the region of interest (ROI) covering the tissue was manually selected for all modalities. For the co-registration process, H&E image was selected as the fixed image and the TPF and SHG images were co-registered among them. To this end, Elastix python library was used.^37^ Deformable co-registration of multi-source images is an ill-posed problem that is hard to be solved appropriately. Additionally, co-registration of images with sizes greater than 50,000 × 50,000 pixels deals to multiple complications. First, the scale of both modalities was homogenized by mapping the manually selected ROIs from SHG and TPF images into the H&E ROI. A second stage comprised a rigid co-registration to align the different image modalities. In order to allow for better computation, the registration map was calculated over a decimated version of the images and then transferred to the original scale images. Finally, a b-spline based deformation map was calculated. Spatial samples were set to 32,000, iterations to 2048 and histogram bins were set to 64. An important hyperparameter is the grid spacing that defines the number of splines used to estimate the deformation that was set to 200. Grid spacing controls the regularization strength to avoid reregistration collapsing into local minima overfitting that deals to unrealistic strong deformations. Functional to measure the difference among both modalities was set to Mattes–Mutual information^30^ as it performs better for co-registration of images from different modalities. Results for this registration are depicted in Fig. 5.

**Fig. 6:**
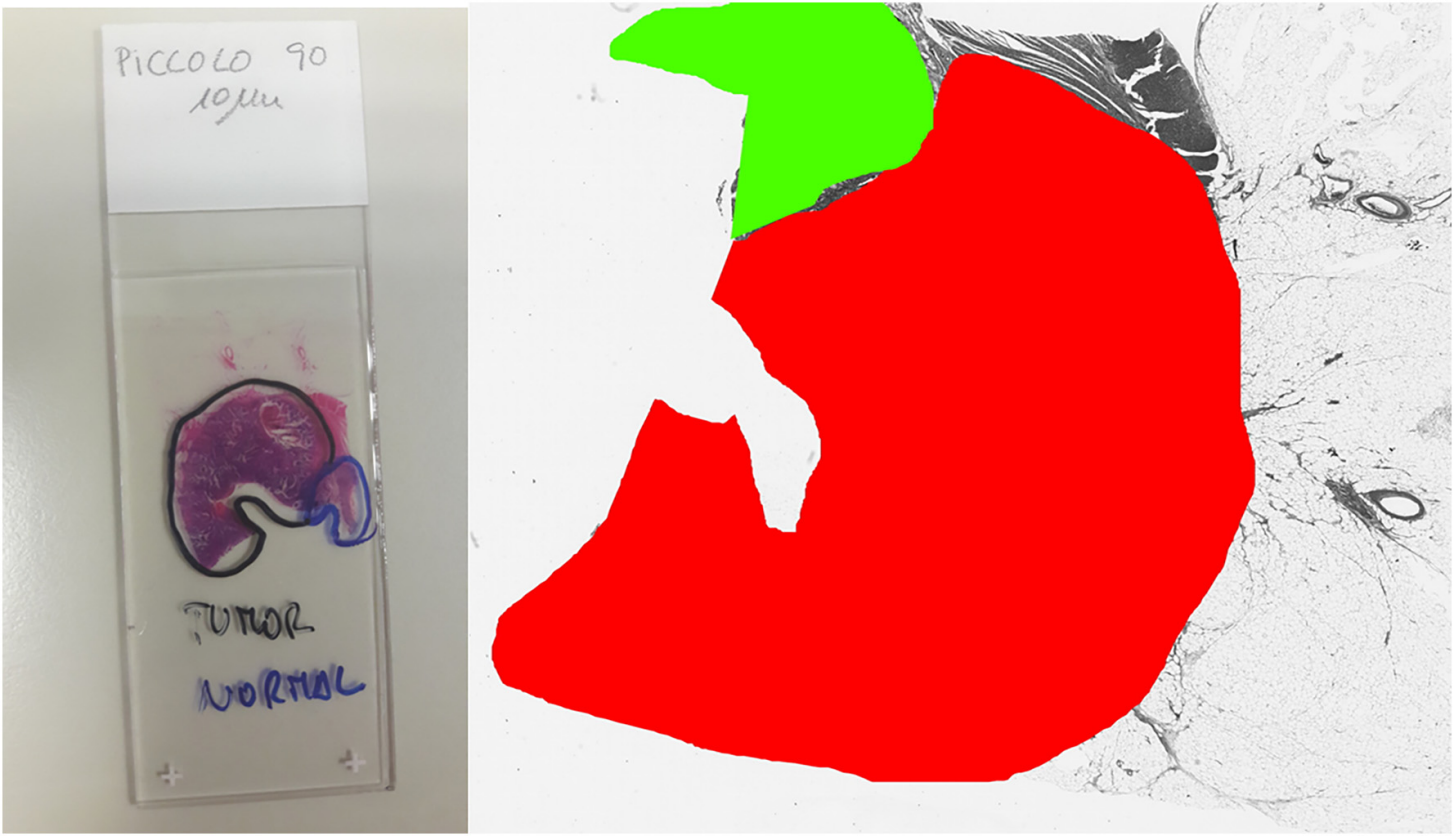
Ground-truth generation. Left) Picture of the microscopy slide. Right) generated ground-truth image.

Annotation of the dataset was performed by trained pathologists. They manually segmented parts of the microscopy slides as healthy or lesion. Lesion parts refer to the lesion described in Table 1. Ground-truth files were created over 1/10 sized images by manually segmenting with green colour (healthy parts) and red colour (lesion parts), as illustrated in Fig. 6.

### Pilot Analysis of the Clinical Semantic Gap Among H&E and MPM Image Modalities

In previous work,^1^ we qualitatively analyzed the clinical capabilities of MPM images where the main conclusion was that, although cellular structure is apparent, the level of detail appreciated on MPM images was felt to be less than that found in traditional H&E slides. Part of the reason for this was the reduced visual contrast, as the MPM images are greyscale as opposed to the coloured H&E staining, as well as the reduced resolution, as the MPM images were acquired with low resolution in order to scan cm^2^-sized samples in a reasonable amount of time. It was also felt that the intracellular features such as cell nuclei were less apparent on the MPM images. Overall, the MPM images do show tissue architecture, but not at the level of detail shown in traditional H&E images which made difficult to make a confident diagnosis.

**Fig. 7:**
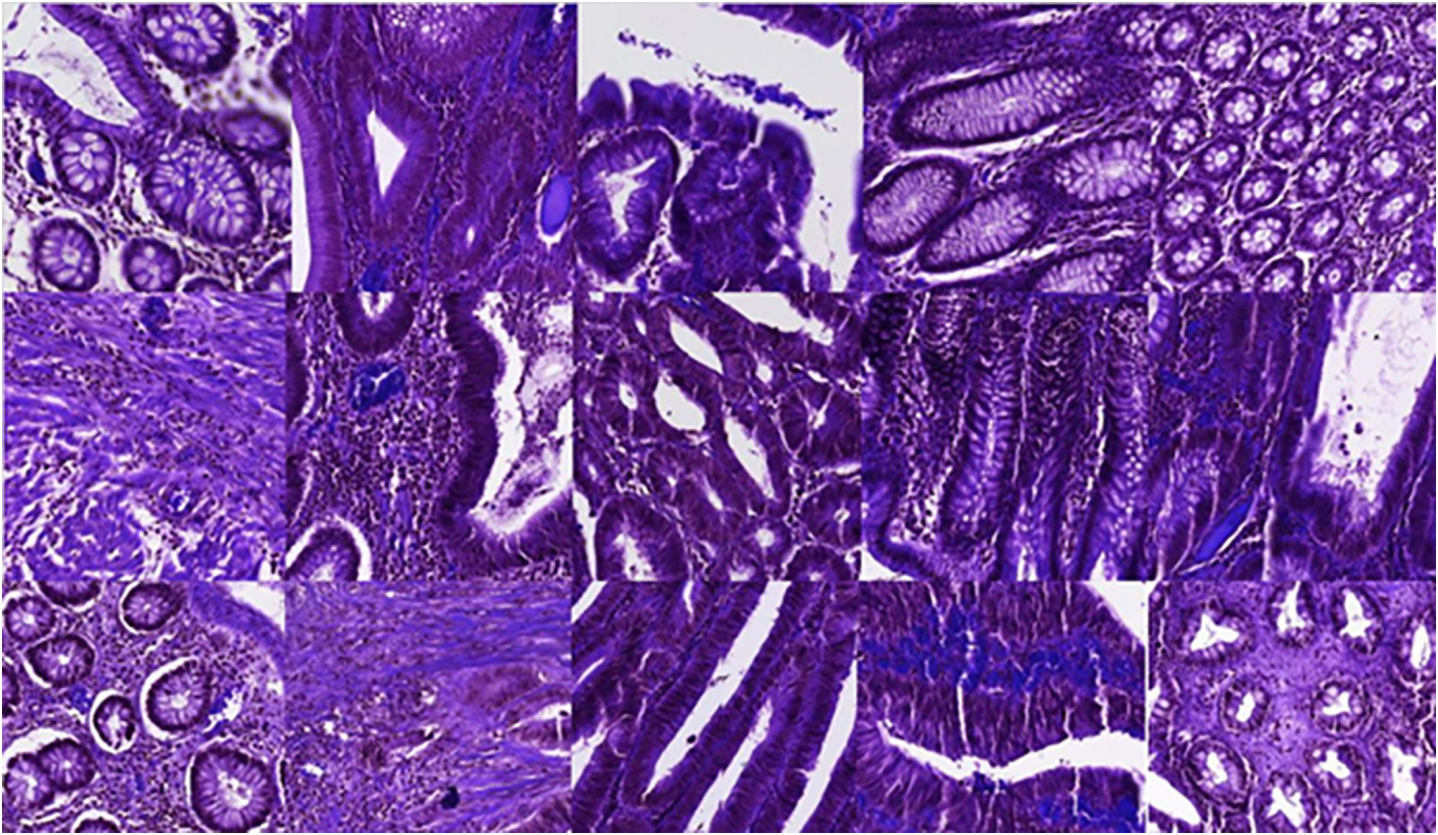
240 × 240 μM tissue parts from different tissue samples. Original H&E images.

**Fig. 8:**
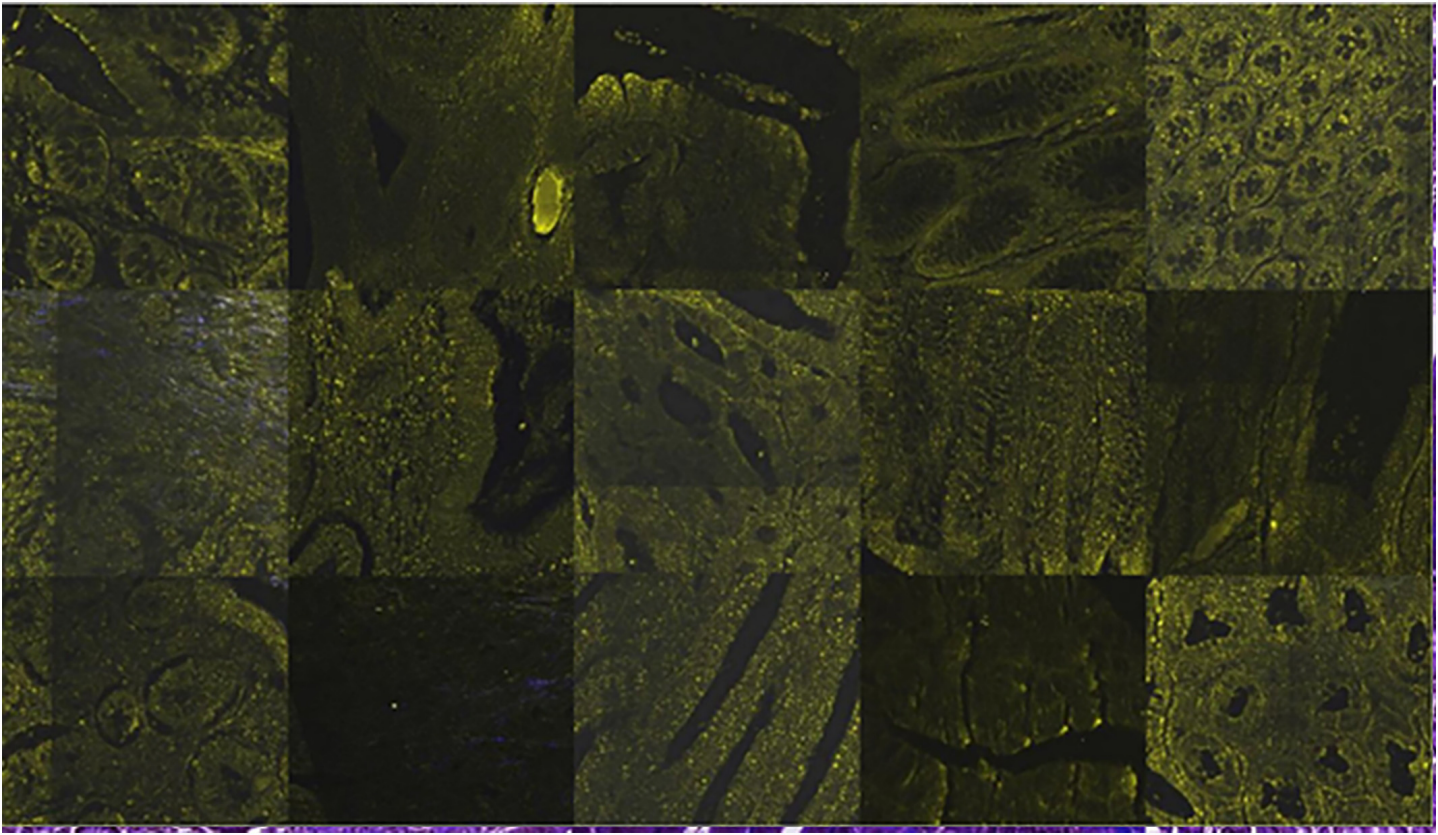
240 × 240 μM tissue parts from different tissue samples. Original TPF images.

In order to preliminarily quantify the diagnostics capabilities ratio between H&E and MPM we have defined a pilot panel composed by five pathologists. To this end, a set of 50 tiles (240 × 240 μM) containing 12 healthy samples, 4 hyperplastic samples, 18 adenoma samples, and 16 adenocarcinoma samples were selected for this study. Selected tiles included representative parts of the tissue. It is noteworthy that we restricted the field of view for the diagnostics on purpose to challenge pathologists to a subtle and complex task. This allows evaluating more efficiently the diagnostic capabilities among image modalities. Selected tissue samples are depicted in Fig. 7, Fig. 8.

**Fig. 9:**
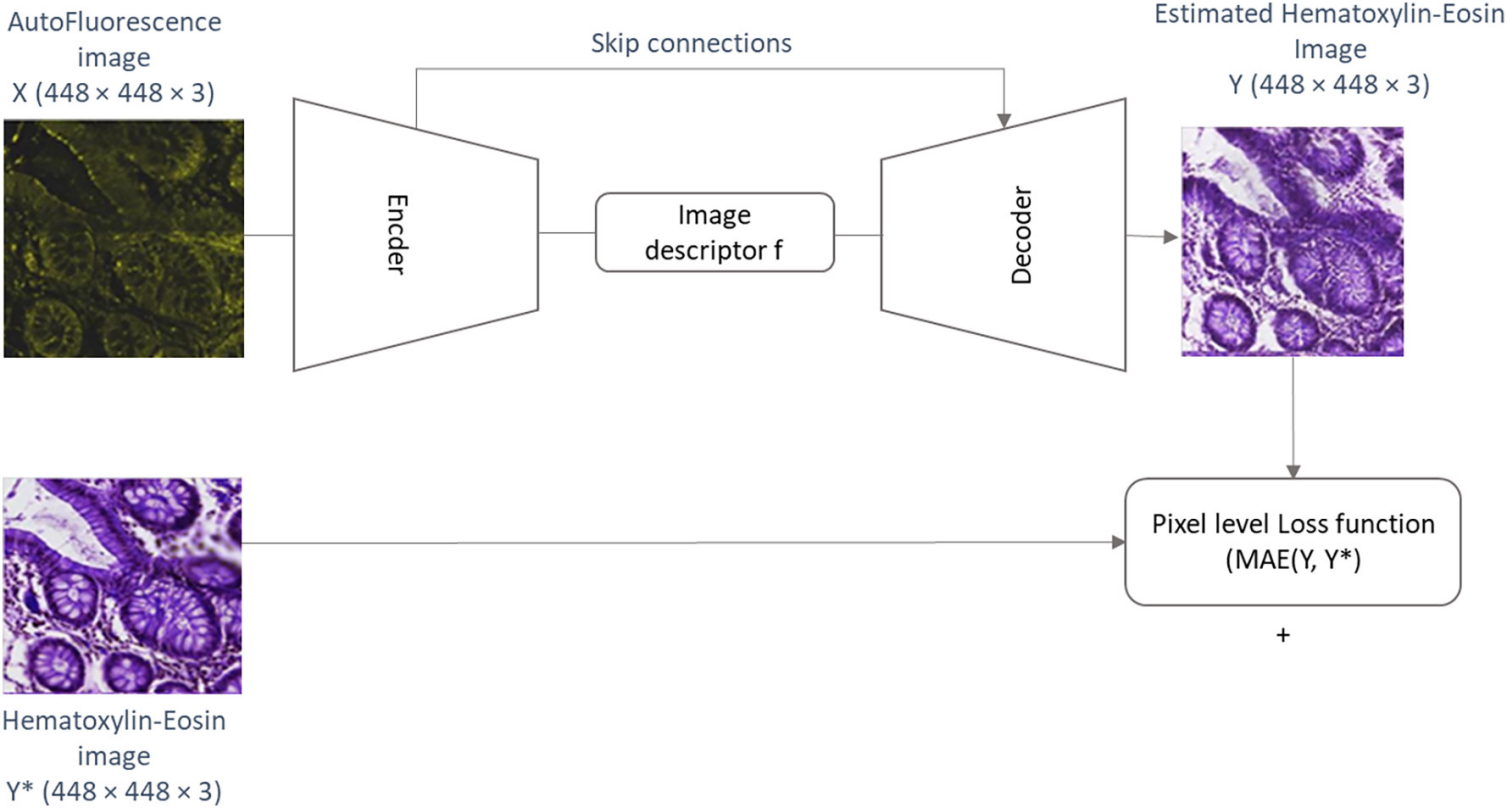
Illustration for the virtual staining architecture. The encoder part receives the input autofluorescence image × and extracts its high-level representation f. The decoder part recovers the input image spatial size to extract the virtually H&E stained estimation Y of the input image X. Training process optimizes the network parameters to minimize the distance between the estimated H&E image Y and the chemically stained H&E image Y*.

**Fig. 10:**
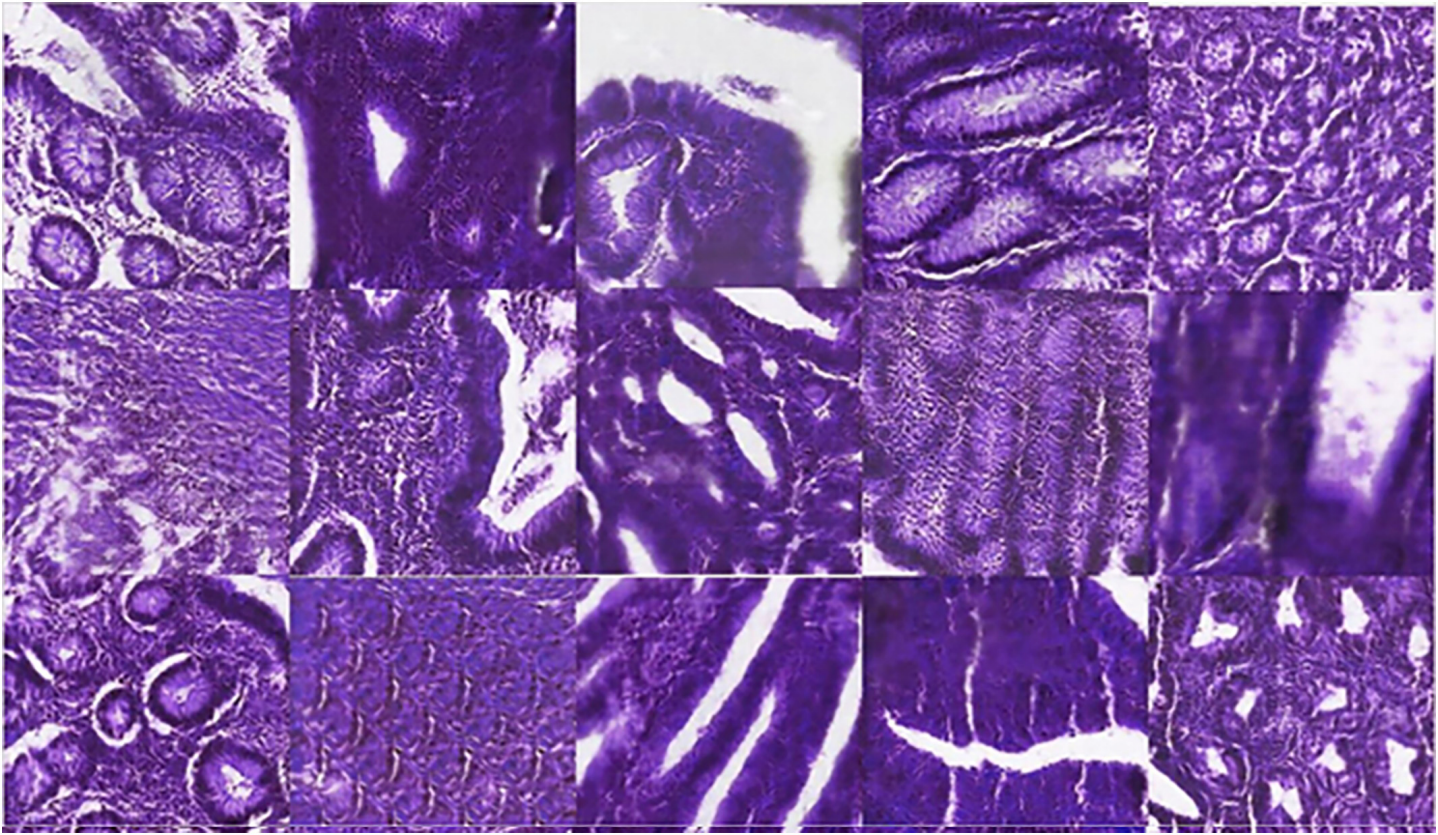
240 × 240 μM tissue parts from different tissue samples. Virtually stained H&E images.

Additionally, we evaluated whether the use of machine learning algorithms that can transform MPM images into virtually stained H&E ones can facilitate diagnosing the images. For that, we have adapted a semantic segmentation network based on a fully convolutional densenet^38^ as depicted in Fig. 9. These networks are comprised by a encoder module that maps the input image × (448 × 448 × 3) through a subsequent set of (learnt) convolutional operations and decimation parts that reduces the spatial dimension of the signal while gradually increasing the descriptive information. After this process, the high-level representation f (7 × 7 × 2048) of the image is obtained. A second decoder module aims to reconstruct the spatial resolution of the original image from the image representation f. To this end, this module is composed by a set of convolutional filters and upsampling layers that recover the spatial resolution of the input image obtaining an output image Y (448 × 448 × 3) that present same dimensions as the input image X. To be able to recover the input image high level details, the network makes use of skip connections^39^ that transfer the low-level features and spatial information from the encoder into the decoder. Final layer of the decoder has been substituted by a sigmoid activation function so that the network can reconstruct the image Y. For the training process, pairs of × (autofluorescence image), Y* (H&E stained image) images are presented to the network. The network is optimized by calculating the network parameters that minimize the mean squared error between the chemically H&E stained image Y* and the virtually H&E stained image predicted by the network Y. Fully technical description of the virtual staining algorithm is provided in ^20^ where we used the baseline Densenet method without embedding regularization. Selected MPM images (Fig. 8) were virtually stained. These virtually stained images are depicted on Fig. 10.

A panel of five pathologists performed the evaluation of the different tiles into four classes (healthy, adenoma/hyperplastic tissue, adenocarcinoma, or unconclusive). F1-score, which is a geometric average among sensitivity and positive-predictive value is used to measure the evaluation. It is noteworthy to highlight that the poor performance even for H&E images is caused by the restriction to a small field of view that precludes pathologists to see contextual information and different scales information. The aim of this pilot is only to preliminarily evaluate the diagnostics performance ratio among modalities.

Table 2 presents the F1 metrics that measure the diagnostics capabilities where we can see that F1 score for H&E images is higher than in MPM modalities (0.33 vs 0.21). This means that MPM performance is 65% of the H&E performance (100%). However, if we employ virtual staining methods, the F1 score for pathologists raises up to 0.30, which is 90% of the H&E performance.

**Table 2:** Dataset histopathological description

Imaging modality	F1 healthy	F1 adenoma/hyperplastic	F1 adenocarcinoma	F1 (average)	% unconclusive	Performance ratio
MPM	0.24	0.29	0.11	0.21	67%	65%
Virtual H&E	0.26	0.36	0.27	0.30	54%	90%
H&E	0.36	0.38	0.25	**0.33**	45%	100%

In addition to the diagnostic accuracy testing performed by the pathologists, a free-form discussion of representative images was conducted to try to further understand the clinical utility and the capabilities of virtually stained images. In particular, feedback was requested regarding the level of detail and features visible in the virtual H+E images. We also enquired regarding the degree of fidelity to original tissue features, and the overall diagnostic confidence the pathologists felt when evaluating the virtual images.

The findings are collated and presented in Table 3. Colonoscopy is the current gold-standard technique for colorectal lesions detection and management. However, this technology cannot assure clear margins and complete resection as histopathology analysis is performed at laboratory. Because of that, novel techniques that can allow in vivo and in situ optical biopsies are being analyzed.

**Table 3:** Virtually H&E stained images findings

Microscopic feature	Virtual stained images
Tissue architecture	The overall tissue structure and architecture was notably similar to the original tissue H+E slides. This could give a reasonable assessment of the tissue at a first glance, for superficial analysis.
Neutrophils	Highly stained cells, such as neutrophils, in the H+E images were far less apparent in the virtual stained images.
Intracellular features	The images were of insufficient magnification to determine intracellular features, but appeared to lack adequate definition to identify intracellular features.
Colonic crypts	Colonic crypt architecture was not visible on the reconstructed autofluorescence images, but reappeared after the virtual staining algorithm. Some slides showed preserved crypt architecture from the original H+E, whereas others showed gross artefact.
Overall degree of image fidelity	The colour and nature of the images broadly resembles the original H+E slides. Many images showed preserved tissue structure, although three were identified as having significant artefact on the virtual staining reconstruction.
Diagnostic confidence	The overall degree of diagnostic confidence was low, due in part to absence of clarity of intracellular features, and partly due to inconsistencies in tissue structure.

MPM has shown potential for gastrointestinal tissue characterization. However, clinicians cannot perform confident diagnosis with this novel modality when using low magnification objective lenses, as in this study, because of a limited spatial resolution. Scanning the samples with higher spatial resolution by means of a high-NA objective could represent a solution to provide the required high spatial resolution for an efficient diagnostic, as demonstrated in ^14^. On the other hand, the acquisition of very large fields of view in the cm^2^ range risks to be impractical because of the extremely long acquisition time required for scanning. Increasing the spatial resolution in specific areas that are crucial for diagnostics or fastening the scanning speed by means of faster scanners or through a multibeam approach could make the difference for the widespread use of MPM technology among pathologists. Anyway, this is beyond the aim of this paper, that is to present and make publicly available a pixelwise co-registered large dataset with H&E, TPF, and SHG imaging modalities that can be employed by the research community to work both on the clinical examination of this technology or the development and comparative analysis of machine learning models among the different modalities.

Additionally, we have validated that diagnostics performance of MPM images under small field of view restrictions is 65% than the performance obtained when looking at the H&E images. When we employ a baseline virtual staining model to transform MPM images into virtual H&E images this performance raises up to 90%. However, virtual staining methods are far for providing confident diagnostics to clinicians and further work might be done.

Future work will include both optical development to get appropriate image quality that could be integrated into functional colonoscopes. Presented dataset is stored by Basque Biobank and request form can be found at: https://www.biobancovasco.org/en/Sample-and-data-e-catalog/Databases/PD181-PICCOLO-EN3.html

## Financial support

This work was supported by the PICCOLO project. This project has received funding from the European Union's Horizon 2020 Research and Innovation Programme under grant agreement No. 732111. The sole responsibility of this publication lies with the authors. The European Union is not responsible for any use that may be made of the information contained therein.

This research has also been supported by the project ONKOTOOLS (KK-2020/00069) funded by the 10.13039/501100003086Basque Government Industry Department under the ELKARTEK program.

## Disclosure

All experiments carried out in this work have been approved by the Basque Clinical Research Ethics Committee (CEIC-E).

## Competing interests

The authors declare that they have no competing interests.
